# HIV-1 integrase resistance in the context of antiretroviral therapy in paediatric patients ‒ Northeast Brazil

**DOI:** 10.1016/j.bjid.2026.105788

**Published:** 2026-02-28

**Authors:** Aldicléya Lima Luz, Élcio Leal, Kledoaldo Lima, Mikhael Morais de Souza, Michelle Lima de Carvalho Silva, Mayra Moura Lima, Paloma Gomes Tavares Sette, Yasmim Leandra Moura de Almeida, Heloísa Ramos Lacerda de Melo

**Affiliations:** a Universidade Federal do Maranhão, Faculdade de Medicina, Imperatriz, MA, Brazil; b Programa de Pós-Graduação em Medicina Tropical, Recife, PE, Brazil; c Universidade Federal do Pará, Instituto de Ciências Biológicas, Laboratório de Diversidade Viral, Belém, PA, Brazil; d Faculdade Pernambucana de Saúde (FPS), Recife, PE, Brazil; e Universidade Federal de Pernambuco, Hospital das Clínicas, Cidade Universitária, Recife, PE, Brazil; f Universidade de Pernambuco, Faculdade de Ciências Médicas, Departamento de Medicina Clínica, Recife, PE, Brazil

**Keywords:** HIV-1, Integrase, Integrase inhibitors, Anti-Retroviral agents, Molecular epidemiology

## Abstract

**Introduction:**

Antiretroviral drug resistance poses a challenge to therapy efficacy. Although Integrase Strand Transfer Inhibitors (INSTIs) have a high genetic barrier to resistance, few studies in Brazil have focused on INSTI resistance. This study aimed to assess HIV mutations associated with INSTI resistance in vertically infected children in Maranhão and Pernambuco, Brazil.

**Methods:**

We conducted a retrospective analysis of antiretroviral resistance profiles in paediatric patients with virological failure receiving care through the Brazilian Unified Health System. Data on viral load, CD4+/CD8+ *T*-cell counts, and medication dispensing were obtained from Ministry of Health databases (SISGENO, SISCEL, and SICLOM). HIV-1 integrase sequences were analysed. Phylogenetic analysis (IQ-TREE) was used to identify subtypes and recombinants. Resistance-associated mutations were determined using the Stanford HIV Drug Resistance Database.

**Results:**

Thirty-one paediatric patients (median age = 12-years) were included. Mean CD4+ count was 999 cells/mm³, and median viral load was 19,235 copies/mL. Subtype B and INSTI resistance mutations were detected in 74.5 % and 64.5 % of patients, respectively. High resistance rates to raltegravir (70.9 %) were observed, highlighting the importance of genotypic monitoring.

**Conclusion:**

A high frequency of INSTI resistance-associated mutations was identified, indicating the need for continued surveillance in this population.

## Introduction

Despite significant advances in Human Immunodeficiency Virus (HIV-1) treatment, antiretroviral drug resistance remains a critical challenge to the efficacy of Antiretroviral Therapy (ART), as it drives the accumulation of resistance-associated mutations, virological failure, and increased morbidity.^1^, ^2^, ^3^, ^4^, ^5^ Integrase Strand Transfer Inhibitors (INSTIs) are a class of antiretrovirals with a high genetic barrier to resistance, potent viral suppression, favourable safety profiles, and fewer drug-drug interactions.^6^ In Brazil, INSTIs are now recommended as the first- and second-line ART regimens following virological failure.^7^^,^^8^ Since 2017, the Brazilian Ministry of Health has adopted the preferred initial regimen of tenofovir disoproxil fumarate, lamivudine (3TC), and Dolutegravir (DTG), a second-generation INSTI.^7^^,^^8^ Recent national guidelines advocate for optimised dual therapy (3TC + DTG), particularly for patients with HIV-associated comorbidities.^9^ By 2020, approximately 410,000 Brazilian patients were receiving INSTI-based regimens.^10^ Given Brazil’s continental-scale implementation of these therapies, the widespread use of INSTIs may accelerate the emergence of resistance-associated mutations, potentially compromising their long-term effectiveness.

Tao et al. (2023) identified 99 sequences with INSTI resistance-associated mutations by analysing antiretroviral resistance profiles.^11^ Scutari et al. (2020) reported an INSTI resistance frequency of 36.4 %, among treatment-experienced individuals and those with long HIV infection duration.^12^ However, other studies have detected INSTI resistance mutations even in treatment-naïve patients.^13^^,^^14^ The identification of antiretroviral resistance mutations is widely recognised as a cost-effective strategy for optimising antiretrovirals and preventing the initiation or continuation of regimens that are likely to result in virological failure, which is a critical measure for HIV epidemiological control. The World Health Organization recommends routine resistance testing in children at diagnosis and after virological failure.^15^ According to Joint United Nations Programme on HIV/AIDS (UNAIDS), approximately one-third of children with HIV infection worldwide experience virological failure within 2-years of ART initiation.^16^, ^17^, ^18^

In Brazil, Ventosa-Cubillo et al. (2023) analysed HIV-1 sequences from 67 children and adolescents and identified INSTI-associated Drug Resistance Mutations (DRMs) in eight patients.^19^ Andrade et al. (2017) studied 117 children with HIV infection (mean age = 3.7-years) in Northern Brazil and reported high DRM frequencies, even among those who were treatment-naïve, particularly for non-nucleoside reverse transcriptase inhibitor-associated mutations.^20^ A retrospective Brazilian study on individuals with virological failure detected INSTI resistance mutations in 22.1 % of participants.^10^ Notably, a large multicentre study of mother-infant pairs in South Africa, Brazil, and Argentina demonstrated a strong association between maternal DRMs and their transmission to infants.^21^

Brazilian studies evaluating INSTI resistance have reported high frequencies of polymorphic variants associated with an increased risk of DRMs.^22^, ^23^, ^24^ However, data on INSTI resistance profiles, specifically in paediatric populations, remain scarce. Given Brazil's early adoption of INSTI-based regimens as first-line ART, understanding the resistance patterns in children is critical. Surveillance of DRM dissemination represents a fundamental strategy for HIV control, particularly in regions such as Brazil, where INSTIs were rapidly integrated into national treatment guidelines. This study aimed to characterise HIV-1 resistance mutations to INSTIs among children who were vertically infected and received care in the Maranhão and Pernambuco states of Northeast Brazil.

## Material and methods

### Study population and setting

This retrospective study evaluated the antiretroviral resistance profiles of paediatric patients diagnosed with HIV who experienced virological failure and were undergoing antiretroviral treatment. The participants were followed by the Unified Health System in the states of Maranhão and Pernambuco in Northeast Brazil. Virological failure was defined as two consecutive detectable viral loads with a minimum interval of 4-weeks (https://www.gov.br/aids/pt-br/central-de-conteudo/pcdts/pcdt_hiv_modulo_1_2024.pdf). The study included children and adolescents (aged < 18-years) with a confirmed HIV infection who had undergone genotyping and sequencing of the HIV-1 integrase region. We obtained 12 sequences from the Maranhão State and 19 from the Pernambuco State, collected between 2018 and 2022.

Data were collected from the Maranhão Central Public Health Laboratory, Maranhão Specialised Healthcare Secretariat, Oswaldo Cruz University Hospital of the University of Pernambuco, and the Brazilian Ministry of Health. Databases containing information on viral load results (SISGENO), CD4+ and CD8+ *T*-lymphocyte counts (SISCEL), and antiretroviral medication dispensing data (SICLOM) were accessed. Data were collected from January to June 2024 using the medical records of patients who were diagnosed with HIV and followed up in outpatient clinics. Information was extracted from the available records of the study sites in the Medical Archives Services. A specific questionnaire was used to collect sociodemographic, laboratory, and clinical variables, which were subsequently analysed using Stata 13.0® software (United States).

### Phylogenetic analysis

HIV-1 query sequences (*n* = 31) were initially analysed using the REGA HIV-1 subtyping tool (version 3.0; http://dbpartners.stanford.edu:8080/RegaSubtyping/stanford-hiv/typingtool/) and COMET HIV-1 for the preliminary viral subtyping classification.^25^ Reference sequences were obtained from the HIV sequence database at the Los Alamos National Laboratory (http://www.hiv.lanl.gov/components/sequence/HIV/search/search.html). Sequence alignments of the study and reference sequences were performed using AliView and manually edited using BioEdit.^26^^,^^27^ Nucleotide substitution models were inferred based on the maximum likelihood and Bayesian information criterion using MEGA software version 11.^28^ A general time-reversible model with gamma distribution and invariant sites (GTR+*G* + *I*) was selected for the alignment analysis. Phylogenetic inferences were made using the maximum likelihood method in IQ-TREE online, with statistical support assessed using 1000 bootstrap replicates.^29^ The resulting phylogenetic trees were visualised using FigTree software version 1.4.3 (http://tree.bio.ed.ac.uk/software/figtree/).

### Antiretroviral resistance analysis

HIV-1 drug resistance mutation analysis was performed by submitting the sequences to the Stanford University HIV Drug Resistance Database (http://hivdb.stanford.edu), which utilises a standardised list of major HIV-1 DRMs from the Stanford HIV Database (https://cms.hivdb.org/prod/downloads/resistance-mutation-handout/resistance-mutation-handout.pdf). Antiretroviral resistance was classified according to the HIV Drug Resistance Database output as susceptible, intermediate (including both low- and intermediate-level resistance), and resistant (high-level resistance).

### Statistical analysis

Data were analysed using the Stata 13.0® software. Medians and Interquartile Ranges (IQR) were determined for continuous variables, whereas categorical variables were expressed as counts and percentages. Statistical analyses involved Chi-Square tests for categorical variables and the Wilcoxon rank-sum test for non-parametric comparisons. The Mann-Whitney *U* test was used to assess the differences between medians. For all tests, the significance level was set at *p* < 0.05.

## Results

**Fig. 1:**
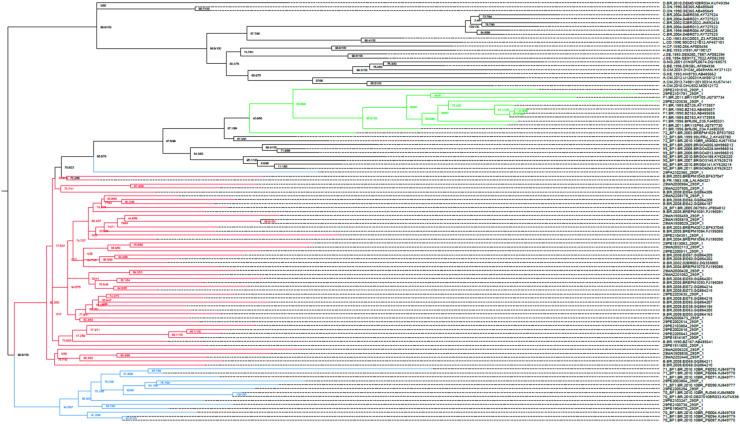
Phylogenetic characterisation of HIV-1 pol sequences obtained from children and adolescents with virological failure in Northeast Brazil. Red: Subtype B; Green: Subtype F1; Blue: BF recombinants.

**Fig. 2:**
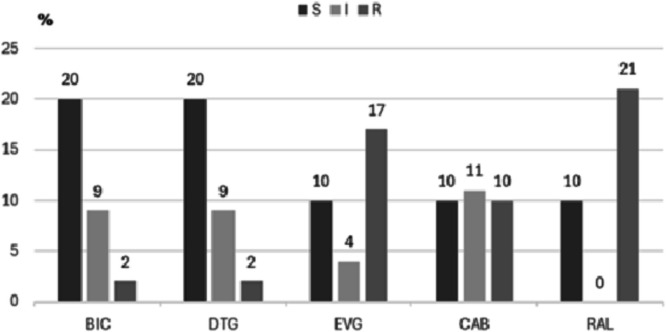
Incidence of antiretroviral resistance to integrase inhibitors in paediatric patients with virological failure in Northeast Brazil. S, Susceptible; I, Intermediate; R, Resistant; BIC, Bictegravir; DTG, Dolutegravir; EVG, Elvitegravir; CAB, Cabotegravir; RAL, Raltegravir.

This study analysed 31 paediatric patients diagnosed with HIV-1 infection who experienced virological failure, with a median age of 12-years (IQR: 9–16). Of them, 18 (58 %) were male patients and 13 (42 %) female patients. The median CD4+ *T*-cell count and viral load at the time of virological failure were 999 cells/mm^3^ (IQR: 707–1432.5) and 19,235 copies/mL (IQR: 1508.5–270,375), respectively. HIV-1 subtyping of the integrase region revealed subtype B (*n* = 23, 74.2 %), BF recombinants (*n* = 5, 16.1 %), and subtype F1 (*n* = 3, 9.7 %) (Fig. 1). Additionally, six patients (19.4 %) had previously used INSTIs (Table 1). Analysis of resistance to INSTIs (Bictegravir [BIC], Dolutegravir [DTG], Elvitegravir [EVG], Cabotegravir [CAB], and Raltegravir [RAL]) showed that 20 sequences (64.5 %) remained susceptible to BIC and DTG, representing the INSTIs with the lowest resistance levels. In contrast, the highest resistance rate was observed for RAL (*n* = 21, 67.8 %) (Fig. 2). Twenty-one HIV-1 sequences (67.8 %) exhibited major INSTI resistance mutations. The most frequent resistance mutations were G140S and Q148H (both *n* = 10, 32.2 %). The Q148K mutation in the integrase gene was observed in 11 patients (35.5 %). Importantly, the Q148H/K mutations conferred resistance to all INSTIs, indicating a high level of resistance in the paediatric population. The N155H mutation was detected in seven patients (22.6 %), of whom two have been receiving antiretroviral therapy that includes INSTIs, and conferred resistance to CAB, EVG, and RAL. Mutations at integrase position 143 (Y143H/R/G) were documented in four patients and conferred resistance to RAL. Among the current ART regimens administered to the patients, the most common were Zidovudine (AZT) + Lamivudine (3TC) + Lopinavir/ritonavir (LPV/r) (*n* = 10; 32.2 %), AZT + 3TC + Nevirapine (NVP) (*n* = 6; 19.4 %), and AZT + 3TC + efavirenz (EFV) (*n* = 5; 16.1 %). The duration of the current ART regimen ranged from 1- to 10-years, with a median of 5.5-years (Table 2).

**Table 1:** Demographic and laboratory characteristics of paediatric patients living with HIV-1 under virological failure ‒ Northeast Brazil.

Characteristics	All patients	Susceptible	Resistance	p-value
	n ( %)	n ( %)	n ( %)	
	31 (100)	10 (35.5)	21 (64.5)	
**Age (years)**				
Median (interquartile range)	12 (9–16)	11 (9.5–14.5)	13.5 (9–16.5)	0.605
**Gender**				
Male	18 (58.1)	4 (36.4)	14 (70.0)	0.074
Female	13 (41.9)	7 (63.6)	6 (30.0)	
**Province**				
Pernambuco	19 (61.3)	9 (81.8)	10 (50.0)	0.086
Maranhão	12 (38.7)	2 (18.2)	10 (50.0)	
**CD4 viral count (cells/mm^3^)**				
Median	999 (707–1432.5)	962 (811–1467)	1064 (422.2–1423.2)	0.695
**Viral load (copies/mL)**				
Median	19,235 (1508.5–270,375)	766 (74.5–149,221.5)	35,950 (6367.8–352,223.5)	0.75
**Subtype HIV-1**				
B	23 (74.2)	6 (60)	17 (81)	0.79
BF	5 (16.1)	4 (40)	1 (4.7)	
F1	3 (9.7)	0 (0.0)	3 (14.3)	
**Use of INIs**				
Yes	6 (19.4)	1 (9.1)	5 (25.0)	0.29
No	25 (80.6)	10 (90.9)	15 (75.0)

**Table 2:** HIV-1 sequences with associated demographic, laboratory, viral subtype, and resistance mutation data.

Patient	Sex	Age (years)	Phylogeny	Current TARV regimen	Time duration of TARV (years)	Mutations	Viral load (copies/mL)	CD4 (cells/mm^3^)
29MA1905459_29	Male	17	B	AZT+3TC+EFV	8	G140S; Q148H	7485	1171
29MA2000420_29	Male	16	B	AZT+3TC+EFV	3	G140S; Q148H	966	3358
29MA2201062_29	Male	17	B	AZT+3TC+NVP	6	G140S; Q148H	2053	222
29MA2000984_29	Male	19	B	AZT+Didanosina+NVP	8	G140S; Q148H	7009	1129
29MA2208178_29	Female	17	B	AZT+3TC+LPV/r	10	Q148K	65,029	999
29MA2006325_29	Female	7	B	AZT+3TC+NVP	2	G140S; Q148H	967,931	901
29MA1909835_29	Female	8	B	AZT+3TC+LPV/r	2	G140S; Q148H	3083,093	1414
29MA2002113_29	Male	10	B	‒	-	N155H	44,086	1559
29MA1905819_29	Female	15	B	AZT+3TC+EFV	5	N155H	27,814	975
29MA2207505_29	Female	3	B	3TC+ABC+RAL	1	SUSCEPTIBLE	5838,695	707
29MA2202440_29	Male	13	B	AZT+3TC+EFV	1	N155H	83,644	13
29MA2000473_29	Male	10	B	AZT+3TC+NVP	5	SUSCEPTIBLE	257,057	785
29PE2101781_29	Male	15	F1	AZT+ 3TC+RAL	3	N155H	372,817	170
29PE2103536_29	Male	10	F1	AZT+3TC+LPV/r	6	N155H	1884	1333
29PE2101510_29	Male	12	F1	3TC+ABC+RAL	3	Y143R	983,811	429
29PE1813063_29	Male	17	B	AZT+3TC+EFV	7	G140S; Q148H; N155H	283,693	545
29PA2102365_29	Female	16	B	AZT+3TC+RAL	‒	Y143G	40	859
29PE2003804_29	Female	11	BF	AZT+3TC+DRV/r	‒	SUSCEPTIBLE	20	1051
29PE2205294_29	Female	15	BF	AZT+3TC+LPV/r	10	Y143H	4444	402
29PE2103804_29	Male	9	B	AZT+3TC+DRV/r	3	G140S; Q148H	371,194	3
29PE2203635_29	Male	9	B	AZT+3TC+LPV/r	5	G140S; Q148H	10,002	3169
29PE1904078_29	Male	9	BF	AZT+3TC+RAL	‒	SUSCEPTIBLE	109	1301
29PE2100738_29	Female	3	BF	AZT+3TC+LPV/r	‒	SUSCEPTIBLE	766	1633
29PE2104351_29	Male	8	B	3TC+ABC+LPV/r	2	G140S; Q148H	2122	1284
29PE2103247_29	Female	11	BF	AZT+3TC+LPV/r	6	SUSCEPTIBLE	1133	865
29PE2200511_29	Female	5	B	AZT+3TC+RAL	‒	N155H	345,900	1451
29PE2002618_29	Male	14	B	AZT+3TC+LPV/r	9	Y143HR	19,235	2635
29PE1814167_29	Female	10	B	AZT+3TC+NVP	‒	SUSCEPTIBLE	241,059	962
29PE2205643_29	Male	12	B	AZT+3TC+NVP	8	SUSCEPTIBLE	20	1661
29PE1911605_29	Male	18	B	AZT+3TC+NVP	8	SUSCEPTIBLE	265	763
29PE2002514_29	Female	20	B	AZT+3TC+LPV/r	9	SUSCEPTIBLE	57,384	707

3TC, Lamivudine; ABC, Abacavir; AZT, Zidovudine; DRV/r, Darunivir/ritonavir; EFV, Efavirenz; LPV/r, Lopinavir/Ritonavir; NVP, Nevirapine; RAL, Raltegravir.

## Discussion

The current study aimed to retrospectively analyse the antiretroviral resistance profiles of INSTIs in virologically failing patients under 16-years of age who were followed by Brazil's Unified Health System. Given the scarcity of research on ART resistance in paediatric populations, our study population selection is a key strength. We analysed 31 HIV-1 integrase region sequences from virologically failing children and adolescents in Northeast Brazil for antiretroviral resistance mutations. Six patients (19.4 %) received INSTIs before genotypic testing for virological failure. The highest resistance frequency was observed for RAL (*n* = 21; 67.8 %), indicating a high-level resistance to at least one INSTI.

The predominant HIV-1 subtype was B (74.2 %), which is consistent with phylogenetic data from previous Brazilian studies that identified this as the most common subtype in both the Northeast and other Brazilian regions, except for Southern Brazil.^29^, ^30^, ^31^ Among the non-B subtypes, BF showed the highest incidence (19 %), aligning with the findings of a previous study conducted in the Maranhão State, Northeast Brazil. However, 2017 data identified HIV-F as the most common subtype in the Pernambuco State, Northeast Brazil,^32^ which contrasts with our findings. The predominance of subtype B may reflect its status as the first subtype to be isolated in the Western world, which facilitated its subsequent spread.

BIC and DTG showed the lowest resistance rates, with 64.5 % of sequences remaining susceptible. Notably, DTG, included in Brazil's first-line adult ART regimen as per the Ministry of Health guidelines, has recently been formulated as dispersible tablets for HIV treatment and prevention in children aged 2-months to 6-years,^33^ significantly improving paediatric treatment feasibility.

Regarding the most prevalent INSTI resistance mutations, in descending order of frequency, we identified G140S, Q148H, N155H, Q148K, Y143H, Y143HR, and Y143R. The high frequency of G140S and Q148H mutations (both *n* = 10; 32.2 %) raises significant concerns, as they confer resistance to all INSTIs. Mutations at positions 140, 143, 148, and 155 of the HIV-1 integrase gene induce resistance to multiple INSTIs.^33^ For instance, N155H (22.6 %, *n* = 7/31) conferred resistance to most INSTIs except DTG. These findings in the paediatric population may suggest consequences associated with prior INSTI exposure, either through the individual’s treatment history or potential maternal transmission. Mutations most associated with INSTI resistance (G140S, Q148H, and N155H) were observed in various combinations among patients infected with subtypes B and F1. Considering the population's median age of 12-years, approximately 35 % showed DTG resistance (only introduced in Brazil's public health system in 2016), and only 19.4 % (*n* = 6/31) had prior INSTI exposure. We hypothesised that INSTI resistance may be emerging rapidly, with treatment adherence likely playing a crucial preventive role.^34^ Mutation distribution analysis revealed that G140S and Q148H frequently co-occurred in patients infected with subtype B virus, whereas N155H appeared in patients infected with subtype B or F1 virus. Patients infected with recombinant BF virus showed INSTI-sensitive profiles, with only one patient exhibiting resistance mutations.

Despite the inclusion criterion adopted in the study, selecting only patients with virological failure who had genotyping results available may have introduced a selection bias towards individuals already suspected of having HIV drug resistance, continuous genotypic monitoring of these patients is essential for therapeutic adjustments to improve clinical outcomes, particularly in paediatric populations. This study highlights the critical need for the surveillance of INSTI resistance mutations, as emerging resistance may compromise treatment efficacy and safety. Systematic resistance monitoring ensures that INSTIs remain a viable therapeutic option, while advancing HIV research to develop more potent treatments and tailored prevention strategies for vulnerable populations. Through these efforts, healthcare systems can implement preventive measures, where early detection of viral resistance may curb the transmission of drug-resistant strains and guide policymakers in optimising antiretroviral selection.

## Ethics approval statement

This study was approved by the Research Ethics Committee of Federal University of Pernambuco, under approval number 5.180.229 (December 20, 2021), in accordance with the guidelines of the National Health Council of Brazil (Resolution 466/12).

## Patient consent statement

The ethics committee accepted the exemption from the patient consent statement, as only HIV-1 medical records and genomic data were obtained.

## Permission to reproduce material from other sources

Permission to reproduce material from other sources was obtained where necessary. All reproduced content is appropriately cited and used with authorization from the original copyright holders.

## Declaration of generative AI and AI-assisted technologies in the writing process

None.

## Funding

This research did not receive any specific grant from funding agencies in the public, commercial, or not-for-profit sectors.

## Data availability

The data that support the findings of this study are available from the corresponding author upon reasonable request.

## Conflicts of interest

The authors declare no conflicts of interest.
